# Transformational Management Properties among Managers of Private and Public Hospitals in Gonbad-e-Kavus City

**DOI:** 10.25122/jml-2018-0066

**Published:** 2020

**Authors:** Jafari Mehrnoosh, Ali Maher, Marziyeh Sheikhi

**Affiliations:** 1. Department of Health Services Management, Tehran North Branch, Faculty of Management,Islamic Azad University, Tehran, Iran; 2. Department Of Health Policy, School of Management And Medical Education,Shahid Beheshti University of Medical Sciences, Tehran, Iran; 3. Department of Health Services Management, Health School, Islamic Azad University of Tehran North Branch, Tehran, Iran

**Keywords:** Transformation management, management properties, charismatic characteristics

## Abstract

Transformational management properties could make the weakest organizations strong with the right and efficient approach build. The aim of this study was to determine the transformational management properties among hospital managers in Gonbad-e-Kavus. In terms of data collection, the present study is an an applied research involving objective and descriptive-analytical surveys. The statistical population includes all staff hospitals that were selected randomly in Gonbad-e-Kavous city. The present study was performed including 7 hospitals and 346 respondents. Castiglion transformational management properties questionnaire (2006) was used to collect the required data, with Cronbach’s alpha coefficient (0.946). Based on the findings of the research, 67.3% (233 subjects) of the participants in the study evaluated the transformational management properties of the managers of the hospitals at a good level (with a score of 140. 76 ± 27.76), with Payambar Hospital having the highest (151.57 ± 20.31) and Taleghani Hospital the lowest score (130.22 ± 28.10) attributed to the management properties affecting change management. Based on the findings of the research and the impact of effective transformational management properties among the managers of the understudy hospitals, it is recommended to form all effective management transformational management properties, through founding special educational units and holding classes for hospital managers, in order to form various skills and competencies such as communication skills, skills to influence others, trusting, attention skills, and providing sufficient independence which all play an essential role in the success of managers and pave the ground for the flourishment of transformation and innovation in the hospitals.

## Introduction

The primary concern of organizations in today’s competitive business environment is to survive and develop as much as possible. In line with this objective, managers are seeking to identify and make optimal use of the resources and capital. The proper implementation of which imposes huge costs and requires serious effort [1]. Managers who make the most effective investments in the most efficient way possible by changing the organization’s management and evolution become the most successful. The evolution of the organization requires a fundamental change in the philosophy of management, education, thinking, culture, and strategies [1, 2]. Considering the importance of this issue in organizations, thinkers and management researchers have sought to discover the characteristics of successful managers in organizations and are constantly trying to express the profile of successful managers in the organization. Thus, various schools of management and theories have been shaped and presented in the scientific community following these efforts in recent years [3-5]. One can refer to the most recent of these perspectives, namely the transformational management properties; this style of management quickly attracted the attention of theorists, thinkers, researchers, brokers, and managers. Therefore, various centers and groups have been set up to elucidate this specific new perspective and make it understandable and applicable for managers [6]. The managers of healthcare organizations must become entrepreneurial and supportive of transformational management properties in order to develop the organization as much as possible [7].

Accordingly, the existence of managerial strategic, or, in other words, transformational management properties is an inevitable necessity [8, 9]. Transformational management properties are effectively trying to use human resources of the organization to achieve organizational goals [10], with preferring efficiency over effectiveness [11, 12]. Duckett and Macfarlane have considered four transformational management properties: individual considerations, inspiration, intellectual stimulation, and charisma, which, from a managerial point of view, are essential to the individual considerations of all employees for the organization who seek to establish personal relationships with the staff [13]. Whether or not charismatic leadership or the ideal effect persuades employees to stick to and follow their leader as a model p14]. This fact that the success of any organization is subject to the guidance and management of dynamics and flexibility has made the role of transformational management properties quite central [15, 16]. Therefore, considering the importance of improving the efficiency and effectiveness of health services, the need to evaluate and review transformational management properties in the managers (clinical and administrative) of hospitals is important in order to identify the strengths and weaknesses of the managers of such organizations to accept the transformation of their organization.

## Material and Methods

The present cross-sectional descriptive study was conducted in all hospitals, emergency departments, and administrative departments of public and private hospitals in Gonbad-e-Kavus city in 2018. The study population included all the staff of hospitals, with the only inclusion criterion being having a diploma degree. In order to select the optimal samples, they were selected randomly according to the number of hospitals and units of management, health departments, nursing and hospital units, management offices, and diagnostic and therapeutic para-clinic and administrative units for each hospital. The sample size was obtained by using the Cochran sample size formula and Morgan’s table. A number of 380 subjects were selected; 380 questionnaires were distributed among the participants, and 346 correct questionnaires were collected. To collect data, the Castiglione transformational standard management questionnaire (2006) which consists of two sections of the individual and demographic information of employees, and questions related to the four main dimensions of the study, including managerial characteristics related to employee charisma (9 questions), managerial attributes related to motivation thought in the staff (10 questions), management properties that reflect individual considerations: (10 questions), managerial properties that facilitate the transformation (10 questions) were used [18]. The questionnaires were answered according to the Likert Scale, with the following response options: I totally agree (5), I agree (4), I neither agree nor disagree (3), I disagree (2), and I and totally disagree (1). Content validity of the questionnaire was obtained using the CVR and CVI Lavasha form from the viewpoint of 10 experts for all questions and the rate turned out to be 0.8. The reliability of the questionnaire was calculated using Cronbach’s alpha (0.946). Multi-Factor Leadership Questionnaire (MLQ) is used to measure transformational leadership with specific dimensions. According to the questionnaire, the answers to the four questions of charismatic management, intellectual motivation, individual considerations, and facilitator of transformation are realized. In this research, ethical considerations such as preservation, retention, honesty and non-bias in concluding and publishing and reporting were taken into consideration in all stages of the research. After data collection and recording on the computer, the data were analyzed using the SPSS 20 software by descriptive statistics and calculating central (mean) and dispersion indices (variance and standard deviation). Data were statistically analyzed using a correlation coefficient, ANOVA and independent samples t-test, and the results are presented below. Finally, a p-value of less than 0.05 was considered statistically significant.

## Results

As seen in Table 1, 167 subjects (48.3%) worked in nursing, anesthesiology and other nursing fields, 65 subjects (18.8%) had paramedical professions, and 103 subjects (29.8%) worked in non-medical fields. According to the table and chart data, among the total number of respondents, 62 (17.9%) were in the hospital, 36 (10.4%) were in intensive care units, 46 (13.3%) were in the emergency department, 72 (20.8%) were in the equivalent in the clinic and para-clinic, and finally, 95 (27.5%) subjects were employed in administrative units (Table 1).

**Table 1:** Frequency distribution of demographic characteristics of field of study, work experience, place of service and previous management history of respondents.

**Variable**		**Frequency**	**Percent**
**Field of study**	Nursing	167	48.3
	Paramedical	65	18.8
	Medical	11	3.2
	Non-medical	103	29.8
**Work experience**	Less than 5 years	142	41
	6-10	74	21.4
	11-15 years	47	31.6
	16-20 years	45	13
	More than 21 years	38	11
**Place of work**	Office works	95	27.5
	Emergency	46	13.3
	Intensive care	36	10.4
	Hospitalization	62	17.9
	Clinic and para-clinic	72	20.8
	Surgery room	35	10.1
**Previous management history**	None	297	85.7
	1-5 years	26	7.5
	More than 5 year	23	6.6

Finally, the analysis of the total points obtained in all of the transformational management properties of hospital managers showed that 67.3% (233) subjects had a good level, 32.1% (111 subjects) had a moderate level and only 0.6% (2 subjects) turned out to have poor transformational management properties. Therefore, the average score of effective management attributes on the transformational management properties in the managers of the hospitals was evaluated at a good level with a score of 140.76 ± 27.76 (Table 2).

**Table 2:** Mean scores of management attributes affecting transformational management properties in the studied hospitals.

**Variable**	**Frequency distribution**									**Level of Management Properties Effective on Transformation Management**	**Mean ± SD**
	**Poor**		**Moderate**		**Good**			**Total**			
	**frequency**	**Percent**	**frequency**	**Percent**	**frequency**	**Percent of each individual hospital**	**Percent out of all**	**frequency**	**Percent**		
**Khatam Al Anbia Hospital**	0	0	100	2.9	22	68.8	6.4	32	9.2	Good	135.91 ± 28.42
**Taleghani Hospital**	0	0	8	2.3	28	77.8	8.1	36	10.4	Good	130.22 ± 28.10
**Shahid Motahari Hospital**	0	0	9	2.6	62	87.3	17.9	71	20.5	Good	136.32 ± 27.58
**Shohada Hospital**	0	0	6	1.7	11	64.7	3.2	17	4.9	Good	144 ± 34.12
**Payambar Hospital**	0	0	26	7.5	35	57.4	10.1	61	17.6	Good	151.57 ± 20.31
**Busky Hospital**	1	0.3	16	4.6	19	52.8	5.5	36	10.4	Good	147.69 ± 26.35
**Borzoi Hospital**	1	0.3	36	10.4	56	60.2	16.2	93	26.9	Good	141.68 ± 30.49
**Total**	2	0.6	111	32.1	233	67.3		346	100	Good	140.76 ± 27.76

A comparison was used in order to investigate and evaluate the importance of each dimension of managerial characteristics affecting transformational management properties in hospital managers. The results of this test indicate that the managers of Payambar Hospital have a better status than other managers in terms of having effective transformational management properties. In contrast, Taleghani Hospital managers have earned the lowest points in the domain of an effective managerial approach to transformational management properties (Table 3).

**Table 3:** Hospital rating based on the average scores obtained.

**Hospital**		**Mean ± SD**	**Hospital rating based on the average scores obtained**
**Payambar Hospital**	Management Indicators Affecting Transformation Management	151.57 ± 20.31	First
**Busky Hospital**		147.69 ± 26.35	Second
**Shohada Hospital**		144 ± 34.12	Third
**Borzoi Hospital**		141.68 ± 30.49	Fourth
**Shahid Motahari Hospital**		136.32 ± 27.58	Fifth
**Khatam Al Anbia Hospital**		135.91 ± 28.42	Sixth
**Taleghani Hospital**		130.22 ± 28.10	Seventh

By examining each dimension of the management properties affecting transformational management properties in the hospitals studied, Table 4 shows that the managers of Payambar Hospital obtain the highest scores.

**Table 4:** Hospital rating based on the mean scores obtained in each dimension of the management properties affecting transformational management properties.

**Hospital**	**Mean ± SD**			
	**Characteristics associated with charismatic management**	**Characteristics associated with intellectual stimulation**	**Characteristics associated with intellectual stimulation**	**Characteristics associated with transformation facilitation**
**Khatam Al Anbia Hospital**	30.35 ± 6.09	35.92 ± 7.84	34.53 ± 8.81	35.10 ± 7.60
**Taleghani Hospital**	29.27 ± 5.83	34.19 ± 7.33	33.34 ± 7.66	33.40 ± 8.42
**Shahid Motahari Hospital**	29.72 ± 6.27	35.14 ± 8.02	35.39 ± 7.59	36.06 ± 7.27
**Shohada Hospital**	30.88 ± 6.37	37.88 ± 9.01	37.35 ± 9.68	37.88 ± 9.79
**Paymbar Hospital**	33.64 ± 4.22	39.98 ± 6.13	39.07 ± 6.42	38.87 ± 5.64
**Busky Hospital**	32.61 ± 5.17	38.55 ± 8.06	38.75 ± 6.93	37.77 ± 7.40
**Borzoi Hospital**	31.65 ± 6.48	36.37 ± 8.45	37.65 ± 8.55	36 ± 8.65

To examine the correlation and significance level between the managerial characteristics associated with transformational management properties in the managers of the hospitals with respect to their normal distribution, according to the slip and elongation test, the Pearson correlation coefficient was used in Table 5. The results of this test indicated that all the characteristics of transformational management properties in the hospital managers (managerial properties associated with employee charisma management, managerial properties associated with intellectual stimulation in the staff, management properties that show individual considerations, and management properties that facilitate communication with each other) are interrelated with one another, with a correlation coefficient of 0.4 (P = 0.005). The highest correlation was between intellectual stimulation of employees with managerial characteristics related to charismatic management of employees, with a correlation coefficient of 0.977 and the statistical significance level of p=0.001 in Shohada Hospital; the lowest correlation was between charismatic management and the management properties facilitating the development of change, with a correlation coefficient of 0.640 and a significance level of p=0.001 (Table 5).

**Table 5:** Relationship between transformational management properties’ with Pearson’s correlation coefficient.

Hospital	Effective managerial properties in transformational management properties		Charismatic properties	Intellectual motivation of employees	Individual consideration	Transformation facilitation
**Khatam Al Anbia Hospital**	charismatic properties	Pearson’s Correlation Coefficient	1	0.821	0.771	0.774
		P value		0.001	0.001	0.001
	Intellectual motivation of employees	Pearson’s Correlation Coefficient	0.821	1	0.888	0.838
		P value	0.001		0.001	0.001
	Individual consideration	Pearson’s Correlation Coefficient	0.771	0.888	1	0.876
		P value	0.001	0.001		0.001
	Transformation facilitation	Pearson’s Correlation Coefficient	0.784	0.838	0.876	1
		P value	0.001	0.001	0.001	0.439
**Taleghani Hospital**	charismatic properties	Pearson’s Correlation Coefficient	1	0.904	0.914	0.884
		P value		0.001	0.001	0.001
	Intellectual motivation of employees	Pearson’s Correlation Coefficient	0.904	1	0.909	0.888
		P value	0.001		0.001	0.001
	Individual consideration	Pearson’s Correlation Coefficient	0.914	0.909	1	0.878
		P value	0.001	0.001		0.001
	Transformation facilitation	Pearson’s Correlation Coefficient	0.884	0.888	0.878	1
		P value	0.001	0.001	0.001	0.310
**Shahid Motahari Hospital**	charismatic properties	Pearson’s Correlation Coefficient	1	0.859	0.737	0.764
		P value		0.001	0.001	0.001
	Intellectual motivation of employees	Pearson’s Correlation Coefficient	0.859	1	0.912	0.916
		P value	0.001		0.001	0.001
	Individual consideration	Pearson’s Correlation Coefficient	0.737	0.912	1	0.917
		P value	0.001	0.001		0.001
	Transformation facilitation	Pearson’s Correlation Coefficient	0.864	0.916	0.917	1
		P value	0.001	0.001	0.001	0.750
**Shohada Hospital**	charismatic properties	Pearson’s Correlation Coefficient	1	0.938	0.926	0.884
		P value		0.001	0.001	0.001
	Intellectual motivation of employees	Pearson’s Correlation Coefficient	0.938	1	0.977	0.954
		P value	0.001		0.001	0.001
	Individual consideration	Pearson’s Correlation Coefficient	0.926	0.977	1	0.958
		P value	0.001	0.001		0.001
	Transformation facilitation	Pearson’s Correlation Coefficient	0.882	0.754	0.958	1
		P value	0.001	0.001	0.001	0.779
**Payambar hospital**	charismatic properties	Pearson’s Correlation Coefficient	1	0.846	0.642	0.640
		P value		0.001	0.001	0.001
	Intellectual motivation of employees	Pearson’s Correlation Coefficient	0.746	1	0.839	0.778
		P value	0.001		0.001	0.001
	Individual consideration	Pearson’s Correlation Coefficient	0.642	0.839	1	0.829
		P value	0.001	0.001		0.001
	Transformation facilitation	Pearson’s Correlation Coefficient	0.640	0.778	0.829	1
		P value	0.01	0.001	0.001	0.237
Busky Hospital	charismatic properties	Pearson’s Correlation Coefficient	1	0.845	0.846	0.873
		P value		0.001	0.001	0.001
	Intellectual motivation of employees	Pearson’s Correlation Coefficient	0.845	1	0.891	0.922
		P value	0.001		0.001	0.001
	Individual consideration	Pearson’s Correlation Coefficient	0.841	0.896	1	0.889
		P value	0.001	0.001		0.001
	Transformation facilitation	Pearson’s Correlation Coefficient	0.873	0.922	0.889	1
		P value	0.001	0.001	0.001	0.113
**Borzoi Hospital**	charismatic properties	Pearson’s Correlation Coefficient	1	0.894	0.802	0.756
		P value		0.001	0.001	0.001
	Intellectual motivation of employees	Pearson’s Correlation Coefficient	0.894	1	0.904	0.896
		P value	0.001		0.001	0.001
	Individual consideration	Pearson’s Correlation Coefficient	0.802	0.904	1	0.907
		P value	0.001	0.001		0.001
	Transformation facilitation	Pearson’s Correlation Coefficient	0.756	0.896	0.907	1
		P value	0.001	0.001	0.001	0.015

There was no significant relationship between the management properties affecting transformational management properties and demographic characteristics of the work history, type of membership, and age groups of staff in the studied hospitals (P> 0.05). 

## Discussion

The results of the research indicate that the least association among the management features is related to the charismatic characteristics of managers. This finding is not consistent with the results of Norshahi’s research, because he had stated that the ideal association (charisma) has the highest score among other components of transformational management properties [17]. In line with this study, the results of Nazem’s study indicated that the charismatic component of managers had the lowest mean among the components of transformational management properties [18]. Charismatic leadership emphasizes the leader’s symbolic behavior, inspirational and dreaming messages, non-verbal communication, the tendency to ideological values, intellectual induction of followers by the director, and also reflects the degree of trust of the followers to the leader and the leader’s expectations of the staff in terms of sacrifice and performance.

However, according to the findings of the present study, the managerial characteristics associated with the charismatic management of the staff have the least impact on the tendency of hospital managers towards transformation, but strengthening managerial characteristics related to the charismatic management in the managers of the hospitals, in particular, Taleghani Hospital (83/5 ± 27.29), which turned out to obtain the lowest average score, can lead to significant changes in the hospital outcomes that require dynamic and extensive adaptation to the environmental conditions and staff expectations of the managers, since the hospital staff gets to prefer the objectives of the hospital over their personal interest only if this specific management mode is implemented.

The second most effective management indicator in transformational management properties from the employee’s point of view is the managerial characteristics that reflect individual considerations. The results of the study led by Duckett and Macfarlane indicated that managerial attributes reflect individual considerations, presenting individual attention, individual behavior, coaching, and advice to employees [13, 14], which requires special attention, given the particular focus of this dimension in transformational management properties.

The results of Nurshahi’s study indicated that there is a positive relationship between the dimension of managerial properties indicating individual consideration and the outcome of the satisfaction of the relationship [17]. This is quite consistent with the findings of previous studies since management properties representing individual consideration include attention to individual needs, the individual’s identity, independence and freedom; the attention of managers and leaders in this category can play an important role in creating transformation in the hospitals under study. Management indicators related to the intellectual stimulation of employees in the hospital managers turned out to be the most significant factors, which is consistent with the results of the research conducted by other authors [10]. When paying attention to the morale and motivation of individuals, as well as attention to people’s beliefs and values, transformational management properties can have a great influence on human management, especially in the management of hospitals and health and medical organizations. The authorities of healthcare organizations and hospitals that follow this style are known as successful managers and have a tremendous impact on their followers.

There was no significant relationship between managerial characteristics affecting transformational management properties and demographic characteristics in terms of work history, type of membership, and age groups of staff in the hospitals, which means that male and female employees have the same perception of management properties. This fact is consistent with the results of some studies and inconsistent with the results of the research by others [19]. In a study, researchers concluded that there had been a significant relationship between the management properties affecting transformation management with the gender of the subjects, meaning that women subjects were more frequently fond of transformational management properties in comparison with men; this suggests that gender can have an impact on transformational management properties indicators. According to another research [16], women tend to have higher morale to their subordinates and their colleagues, especially in their nursing and care departments, which is, by itself, a transformational management properties feature. Previous studies also stated that women are more likely to adopt transformational management properties than men. Gender can be an important factor in applying managerial styles. Women tend to manage emotionally and morally because of different mental characteristics than men, which is a feature of the transformational management properties style; this discrepancy between the present research and the results of previous studies can be attributed to the difference in the geographical environment and socio-cultural characteristics of the study population. The findings of the research showed that there is no significant difference between the transformational management properties indicators and the educational level of the staff. The results of this study are consistent with the results of the research conducted by others [19].

It seems that having a high degree is not a reason to choose a creative management style. Employees have used their experiences in determining the impact of transformational management properties indicators and having high qualifications cannot fit into the use of transformational management properties. However, the results of the research are not consistent with the findings of previous studies because the researchers concluded that there is a positive and significant relationship between education with transformational management properties and creativity; thus, the higher the level of education, the higher the score of transformational management properties goes and the more the creativity of the staff gets [12]. However, there turned out to be a statistically significant difference between work experience and management attributes affecting transformational management properties, which turned out to be inconsistent with the results of some studies [20]. According to the researchers, managers who spend more time to get familiar with issues and problems use different strategies and methods to overcome obstacles and problems, something which is not possible without innovation and transformation.

Other results of the research showed a significant relationship between managerial characteristics affecting transformational management properties and the status of the relationship of the staff (p = 0.006); this is not consistent with the results of the research by others, in which there was no significant difference between the transformational style of creative management based on the employment status [1, 10]. 

In general, employment status can be effective regarding transformational management properties. The more secure and stable the job status of the staff is, the more comfortable they perform their task, and the more positive they think to improve the organization’s position, which leads to creativity, innovation, and, consequently, transformational management properties. The main factor in transformational management properties is the charismatic property of managers, which can result in the increase of employees’ motivation, their alignment with the manager, the creation of coherence among the members of the working groups, and the increase of self-esteem and self-efficacy of the staff [17]. Managers who support transformational management properties can dictate their thoughts over the staff and push them beyond their own personal interests by changing their ideals, interests and values, resulting in more efficient feedback than what they expect their staff. The lack of feeling the need to change has always been one of the problems of the hospital management systems in Iran [21].

## Conclusions

Regarding the important effect of managerial properties affecting transformational management properties, related to the intellectual stimulation of employees and individual considerations on various outcomes, in health organizations, the managers and officials of hospitals should not disregard such a vital fact. They, rather, must spare no effort to create job satisfaction in the staff in order to achieve the goal of transformation through appropriate planning and creating better conditions for employees and paying attention to individual needs. Therefore, attempts must be made to form all effective management transformational management properties. These attempts include management properties associated with charismatic and intellectual impetus, management capabilities representing individual aspects and properties of the administrative facilitator of change, through founding special educational units and holding classes for hospital managers in order to form various skills and competencies such as communication skills, skills to influence others, trusting, attention skills, and providing sufficient independence, which play an important role in the success of managers and pave the ground for the flourishment of transformation and innovation in the hospitals.

## Acknowledgment

The present paper is extracted from the master’s thesis of Ms. Marziyeh Sheikhi.

## Conflict of Interest

The authors declare that there is no conflict of interest.
